# A Broad Range Triboelectric Stiffness Sensor for Variable Inclusions Recognition

**DOI:** 10.1007/s40820-023-01201-7

**Published:** 2023-10-20

**Authors:** Ziyi Zhao, Zhentan Quan, Huaze Tang, Qinghao Xu, Hongfa Zhao, Zihan Wang, Ziwu Song, Shoujie Li, Ishara Dharmasena, Changsheng Wu, Wenbo Ding

**Affiliations:** 1 Tsinghua-Berkeley Shenzhen Institute, Institute of Data and Information, Shenzhen International Graduate School, Tsinghua University, Shenzhen, 518055 People’s Republic of China; 2 Institute of Ocean Engineering, Shenzhen International Graduate School, Tsinghua University, Shenzhen, 518055 People’s Republic of China; 3 Wolfson School of Mechanical Electrical and Manufacturing Engineering, Loughborough University, Loughborough, LE11 3TU UK; 4 Department of Materials Science and Engineering, National University of Singapore, Singapore, 117575 Singapore; 5 RISC-V International Open Source Laboratory, 518055 Shenzhen, People’s Republic of China

**Keywords:** Stiffness sensor, Decoupling method, Heterogeneous stiffness, Variable inclusions, Healthcare applications

## Abstract

With the development of artificial intelligence, stiffness sensors are extensively utilized in various fields, and their integration with robots for automated palpation has gained significant attention. This study presents a broad range self-powered stiffness sensor based on the triboelectric nanogenerator (Stiff-TENG) for variable inclusions in soft objects detection. The Stiff-TENG employs a stacked structure comprising an indium tin oxide film, an elastic sponge, a fluorinated ethylene propylene film with a conductive ink electrode, and two acrylic pieces with a shielding layer. Through the decoupling method, the Stiff-TENG achieves stiffness detection of objects within 1.0 s. The output performance and characteristics of the TENG for different stiffness objects under 4 mm displacement are analyzed. The Stiff-TENG is successfully used to detect the heterogeneous stiffness structures, enabling effective recognition of variable inclusions in soft object, reaching a recognition accuracy of 99.7%. Furthermore, its adaptability makes it well-suited for the detection of pathological conditions within the human body, as pathological tissues often exhibit changes in the stiffness of internal organs. This research highlights the innovative applications of TENG and thereby showcases its immense potential in healthcare applications such as palpation which assesses pathological conditions based on organ stiffness.

**Supplementary Information:**

The online version contains supplementary material available at 10.1007/s40820-023-01201-7.

## Highlights

- We propose a broad range triboelectric sensor system employing elastic sponge and shielding layers, which can realize fast stiffness recognition within 1.0 s at a low cost.
- A novel algorithm is proposed for rapid stiffness identification by extracting signal characteristics, effectively reducing demand of computing resources.
- The proposed sensor system can identify the multi-layer stiffness structure of objects, enabling effective recognition of variable inclusions in soft objects with an accuracy of 99.7%.

**Supplementary Information:**

The online version contains supplementary material available at 10.1007/s40820-023-01201-7.

## Graphical abstract

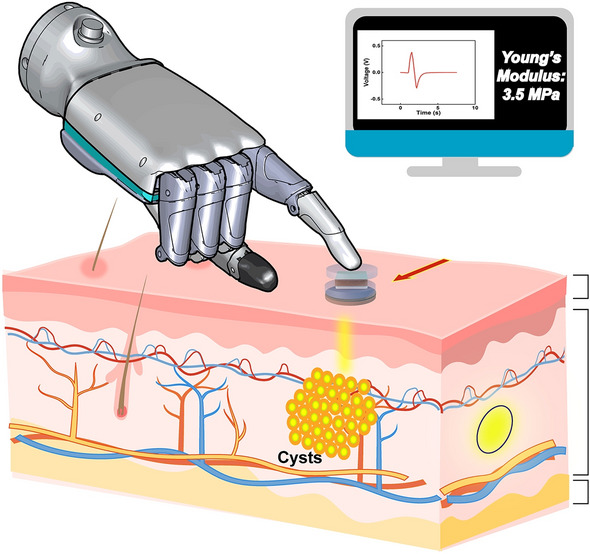

## Introduction

The rapid advancement of artificial intelligence paves the way for robots to be extensively applied in a multitude of fields bringing tremendous convenience to humanity [1–7]. For example, robots assist in palpation with feedback sensors to provide diagnostic results beyond the scope of vision, which necessitates the perceptual capability of robots [8–10]. Specifically, robots equipped with stiffness perception can identify internal structures with varying levels of stiffness, enabling the assessment of a patient's condition based on organ stiffness [11]. However, conventional benchtop stiffness sensing methods utilize force sensors and displacement feedback systems to measure Young's modulus representing object stiffness, which fails to meet the flexible requirements of diverse scenarios [12].

To address the flexibility problem, several signal processing techniques and advanced mechanical designs are proposed. Novel stiffness measuring methodology utilizes multi-sensory electronic skins and deep learning algorithms to avoid the limitation of benchtop [13–17]. However, the algorithms lack interpretability, which limits the adaptation to objects out of training dataset [18, 19]. Nevertheless, to surmount the intricacies associated with the interpretability problem, stiffness sensors that incorporate self-locking structure are proposed [20–22]. The design allows for enhanced control over the sensor deformation and effectively avoids the reliance on displacement feedback systems. In spite of that, the self-locking structure lacks the capability to identify deep-seated objects due to the restrictive design. In details, the sensor reaches a locked state even when subjected to minimal deformation during pressing. This implies that the sensor cannot undergo more significant deformation as the pressing continues, thereby failing to generate corresponding signals. Triboelectric nanogenerators (TENGs) [23–27] can convert mechanical energy into electrical energy based on triboelectric induction and electrostatic equilibrium [28–32]. It has the advantages such as low-cost, simple structure and excellent output performance even at low frequency [33–37]. Consequently, it is suitable for sensing various physical properties such as acceleration [38, 39], wind speed [40, 41], vibration frequency [42, 43], and force [44–48], thereby presenting great potential for stiffness identification. By employing novel TENG structural designs, it becomes possible for the pressing signal to capture information regarding both object deformation and applied force, eliminating the need for restrictive self-locking structures. Additionally, the deformable design of the TENG enables a larger measurement range, overcoming limitations in deep-seated object detection.

In this study, we propose a stiffness sensor based on the triboelectric nanogenerator (Stiff-TENG), overcoming the limitations of previous work [20–22] in adaptable recognition of multi-level objects. The triboelectric layer consists of a fluorinated ethylene propylene (FEP) film coated with conductive ink printed electrode and an indium tin oxide (ITO) layer. When the two layers are forced to approach, charge transfer is introduced and the signal is generated [49]. Through the decoupling of signals, both object deformation and the applied force can be obtained to recognize the stiffness within 1.0 s. The Stiff-TENG demonstrates a substantial measurement range while proficiently detecting stiffness. Benefited from feature engineering and machine learning [50], the Stiff-TENG can recognize the inner structure properties of the multi-level objects, including shape, size, amount, and stiffness of the encapsulated materials. The recognition accuracy can achieve 99.7%. The adaptability makes it possible for the Stiff-TENG to be applied in the detection of pathological conditions within the human body, as pathological tissues usually manifest as alterations of internal organs. Therefore, the Stiff-TENG can serve as the tool for palpation. This research demonstrates the progress in innovative applications of TENG and contains substantial potential for stiffness recognition and healthcare applications.

## Experimental Section

### Materials

The inner structure of Stiff-TENG consists of an ITO film, an FEP film with a conductive ink printed electrode, and an elastic sponge. The FEP film actes as the electronegative triboelectric layer, which has a working area of 10 mm × 10 mm and a thickness of 0.05 mm. Since FEP is an electrically insulating material, a screen-printing device is utilized to print the conductive ink on the backside of the FEP film to transfer electrons between the two triboelectric layers [51] (the fabrication details are illustrated in Fig. S1). The ITO film membraned with PET substrates is utilized as the electropositive layer with the size of 10 mm × 10 mm × 0.2 mm. The hollow elastic sponge is placed between the FEP film and the ITO film to provide enough deformation space, and its size is 10 mm × 10 mm × 2 mm, with a uniform hole (6 mm × 6 mm × 2 mm) inside. The protection structure consists of two circular-shaped acrylic pieces with a radius of 8.5 mm. One acrylic piece is fixed above the ITO film, while the other acrylic piece is adhered to the underside of the FEP film using Kapton. This arrangement provides physical protection for the films. The shielding film with the radius of 8.5 mm is attached to the outer acrylic which is closer to the pressed objects. It is connected to the ground via one shielding line stuck on it. The uneven sponge with a thickness of 1 mm and a diameter of 8.5 mm is attached to the surface of the shielding film.

### Experimental Equipment

For standardized experimental characterization, the Stiff-TENG is mounted on the end tip of the push–pull force gage ZhiQu (ZQ-990LB). During each pressing and releasing, the movement of the equipment tip keeps a constant speed and moves in a straight line. In the experiments below, pressing speed of 150 mm min^−1^ is employed. For the encapsulated object identification utilization, the Stiff-TENG is attached to the Robot arm UR5 via a connection structure printed by 3D printer (Raise 3D Pro2 Plus). All the electric output in the experiments is measured by the electrometer Keithley 6514. The real-time signals measured were pre-processed and displayed on the MATLAB interface in LabVIEW.

## Results and Discussion

### Encapsulated Object and Stiffness Detection System

**Fig. 1:**
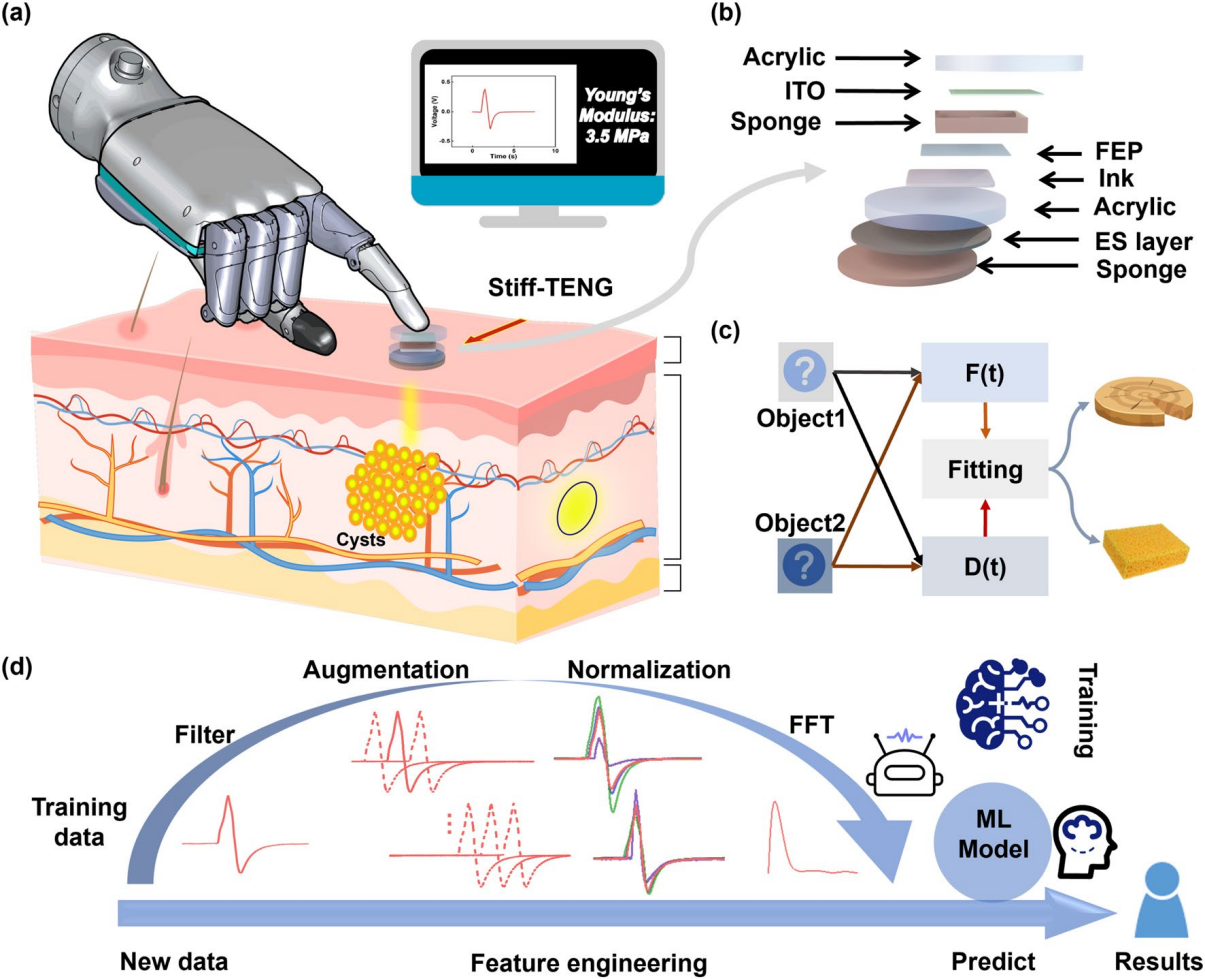
Structure and application illustration of the Stiff-TENG. **a** Schematic diagram of the inclusions recognition application based on the Stiff-TENG. **b** Layered illustration of the proposed Stiff-TENG. **c** Identifying object stiffness by extracting force and displacement information from the generated signals. **d** System-level flow chart of the variable inclusions recognition processing

The detection platform is designed for standard stiffness estimation experiments. The Stiff-TENG is mounted on the end of a vertical push–pull force gage. The Stiff-TENG's two electrodes are connected to a 1 GΩ resistor in series, and linked to the two terminals of the electrometer. When the Stiff-TENG contacts the objects, the mechanical energy is converted into the electrical signal displayed on the computer screen (Fig. 1a). The multi-layer structure of the Stiff-TENG is depicted in Fig. 1b. The effective working part comprises three components. The first is an ITO film, serving as both the tribo-charge layer and the electrode. The second is an FEP film coated with conductive ink printed electrode, functioning as another tribo-charge layer due to its strong electro-negativity. The third component is a holed elastic sponge that allows for sufficient relative displacement between the two tribo-charge layers to enable force measurement over a wide range. Two acrylic pieces are attached to the FEP and ITO films respectively, serving as protective structures. A shielding film is then applied to the bottom and connected to the ground, which aims to prevent the charges on the pressed objects from interfering with the measurement results (Note S1). As shown in Fig. S2, the sensor without a shielding layer generates induced noise while approaching an object. However, the Stiff-TENG with the shielding structure effectively mitigates the interference from surface charges on the objects. To reduce the influence of the pressed object’s surface viscosity, an uneven sponge is attached to the shielding film which can reduce the contact area between the Stiff-TENG and the object. Consequently, the signals generated by the Stiff-TENG during pressing against an object encompass the information of both the applied force and the object deformation which enables the identification of stiffness (Fig. 1c). Therefore, the Stiff-TENG can be deployed on any equipment end to identify stiffness without additional displacement feedback system. When employed for identifying unknown objects encapsulated in a soft structure, the measured signals comprise both the stiffness information and environmental noise. Figure 1d provides an overview of the process flow of data, where a 40 Hz low-pass filter is utilized to remove the environment noise. Subsequently, with the aid of data augmentation, normalization, fast Fourier transform (FFT), and machine learning, the shape, size, amount, and stiffness of the encapsulated objects can be identified.

### Working Principle and Characteristic of the Stiff-TENG for Objects with Different Stiffness

Figure S3a demonstrates the utilization of a vertical push–pull force gage to analyze the performance and characteristics of the Stiff-TENG. The Stiff-TENG is mounted on the equipment end which can move over a fixed displacement at a constant speed after contacting objects (Fig. S3b). The output performance is investigated with various connected external load resistance ranging from 100 MΩ to 10 GΩ (Fig. S4), and the maximum power occurs when the load resistor is 800 MΩ.

**Fig. 2:**
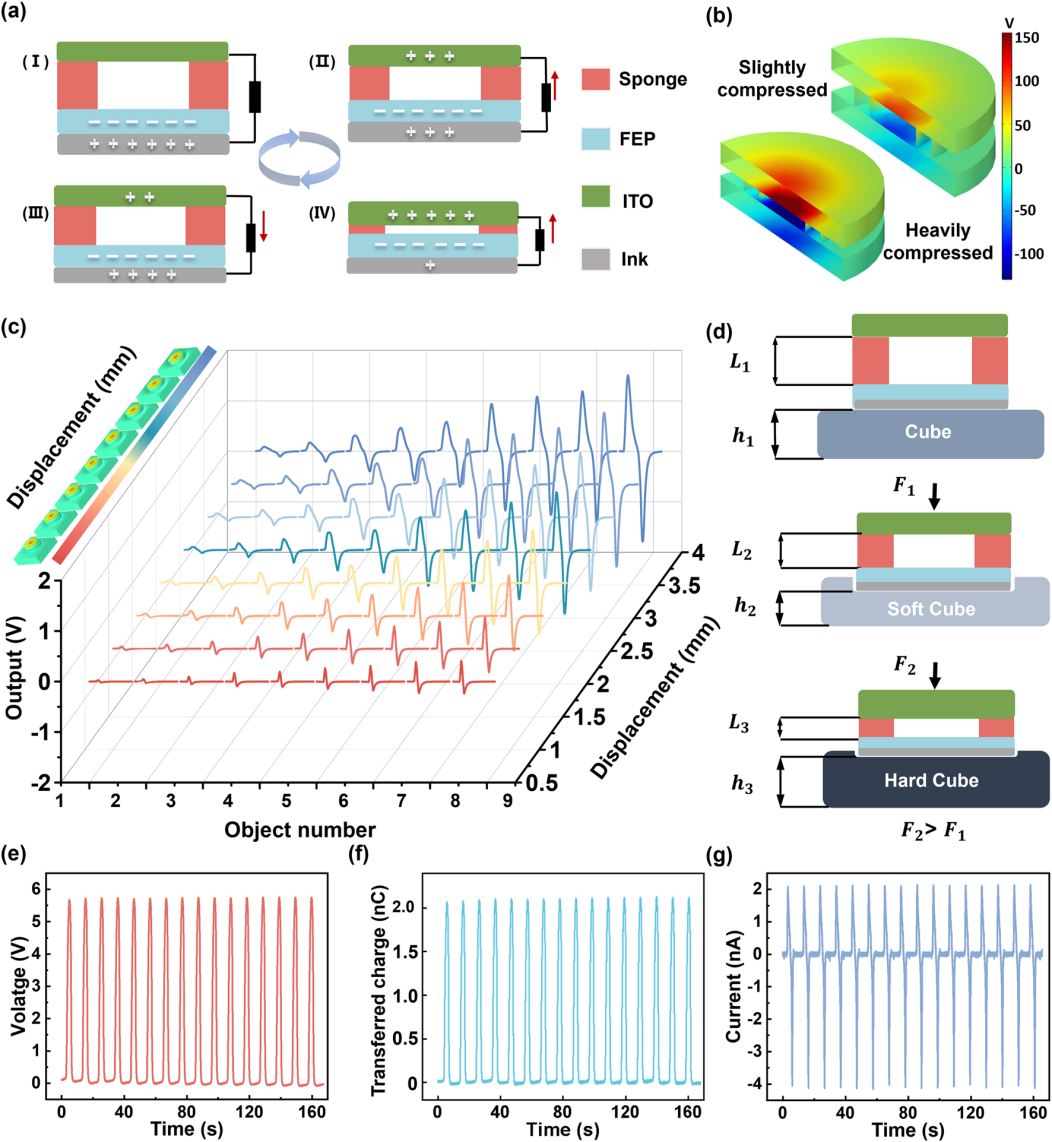
Working principle and characterization of the Stiff-TENG. **a** Working principle of the Stiff-TENG. **b** COMSOL simulation of different compressing level between the two electrodes of the Stiff-TENG. **c** Measured voltage output of Stiff-TENG over nine different stiffness objects. **d** Deformation situation of the sponge and the cube when TENG compresses the soft/hard under the same displacement. **e** Open-circuit voltage of the Stiff-TENG. **f** Transferred charge of the Stiff-TENG. **g** Short-circuit current of the Stiff-TENG

The electric output is generated through the electrostatic induction and electrostatic equilibrium. The working mechanism of the presented Stiff-TENG is shown in Fig. 2a to illustrate the relationship between the deformation of the Stiff-TENG and the output signal. Initially, the charge distribution on the FEP film and the ITO film is uniform, and they are in a state of electrostatic equilibrium. FEP and ITO have different electron affinities. FEP has a stronger electronegativity, which gives it a greater ability to gain electrons. When the Stiff-TENG is compressed, the ITO film and FEP film approach/contact each other. During this process, the electrons on the ITO film redistribute, resulting in a current flowing from the external circuit to the ITO film. As the external force is gradually removed, the sponge-shaped structure recovers, causing the ITO film and FEP film to separate. This leads to a reverse process compared to the compression process, resulting in a reversal of the current direction. Eventually, the layers return to their initial positions, and the device repeats this process when subjected to external forces. Afterward, to explain the relationship between Stiff-TENG deformation and voltage output, assume that the Stiff-TENG is a linear elastic object with Young's modulus of ${\varepsilon }_{1}$. With the surface charge density of the dielectric layer $\sigma$, the Stiff-TENG deformation ${x}$, the air permittivity ${\varepsilon }_{0}$, and the original spacing of the dielectric layer ${d}_{0}$, the voltage $V$ can be expressed by:

1 $$V=\frac{\sigma {d}_{0}}{{\varepsilon }_{0}}-\frac{\sigma ({d}_{0}-{x})}{{\varepsilon }_{0}}=\frac{\sigma {x}}{{\varepsilon }_{0}}$$

Consequently, larger Stiff-TENG deformation results in higher voltage output, which corresponds to the COMSOL simulation result as shown in Fig. 2b.

To investigate the impact of objects with varying stiffness on Stiff-TENG output, nine standard 50 mm × 50 mm × 50 mm silicon rubber cubes (shown in Fig. S5, fabrication details are illustrated in Note S2) are fabricated. They are with different Young’s modulus (0.37, 0.65, 1.23, 1.65, 2.05, 3.70, 4.95, 5.56 and 8.28 MPa), and defined as Object No. 1–9, respectively. The cubes are pressed by the Stiff-TENG along with the movement of equipment tip to deform. The voltage signal under various displacement is shown in Fig. 2c. The corresponding repeating signals are displayed in Fig. S6-S8. To characterize the output performance, the open-circuit voltage *V*_oc_, the transferred charge *Q*_*sc*_, and the short-circuit current *I*_sc_ are measured for Object 9 under 4 mm displacement (Fig. 2e-g). During the pressing process, the hollow elastic sponge of the Stiff-TENG undergoes compression along with the vertical deformation of the pressed cube. The COMSOL simulation depicting the pressing process is shown in Fig. S9 and Movie S1. The total displacement observed is the combined sum of the vertical deformation of Stiff-TENG and the cube. Consequently, the mechanical model is established for the interaction between the Stiff-TENG and the cubes. In the mechanical model, it is assumed that both the Stiff-TENG and the objects exhibit linear elastic behavior, with the Stiff-TENG having Young's modulus of ${\varepsilon }_{1}$ and the objects having Young's modulus of ${\varepsilon }_{2}$. The applied force causes vertical deformation $x$ and $d$ for the Stiff-TENG and the object, respectively, and total deformation is $\widehat{x}=x+d$. Specifically, as shown in Fig. 2d, for the soft object and the hard object, ${x}_{1}={L}_{1}-{L}_{2}$, ${x}_{2}={L}_{1}-{L}_{3}$, ${d}_{1}={h}_{1}-{h}_{2}$, ${d}_{2}={h}_{1}-{h}_{3}$, and ${x}_{1}+{d}_{1}={x}_{2}+{d}_{2}$. According to the constitutive model of linear elastic materials, we have that ${\varepsilon }_{1}x={\varepsilon }_{2}d$. Therefore, under the same total displacement, the relationship between the Stiff-TENG deformation and object stiffness is:

2 $$\frac{{\varepsilon }_{1}}{{\varepsilon }_{2}}=\frac{x +d}{x} - 1$$

It should be noted that the stiffness of the Stiff-TENG remains constant. According to Eq. (2), under the same total displacement, there is a positive correlation between $1/\varepsilon _{2}$ and $1/x$ for the pressed cubes with different stiffness. Therefore, when the measured object has a larger stiffness, the Stiff-TENG undergoes a larger degree of deformation. As a result, based on Eq. (1), the voltage generated by the Stiff-TENG increases as the pressed object becomes harder. The corresponding simulation results are shown in Fig. S10. Under the same total displacement, a harder object corresponds to a larger Stiff-TENG compression and lower cube deformation. At the same time, the predicted deformation of the pressed objects which cannot be directly measured is shown in Fig. S11.

**Fig. 3:**
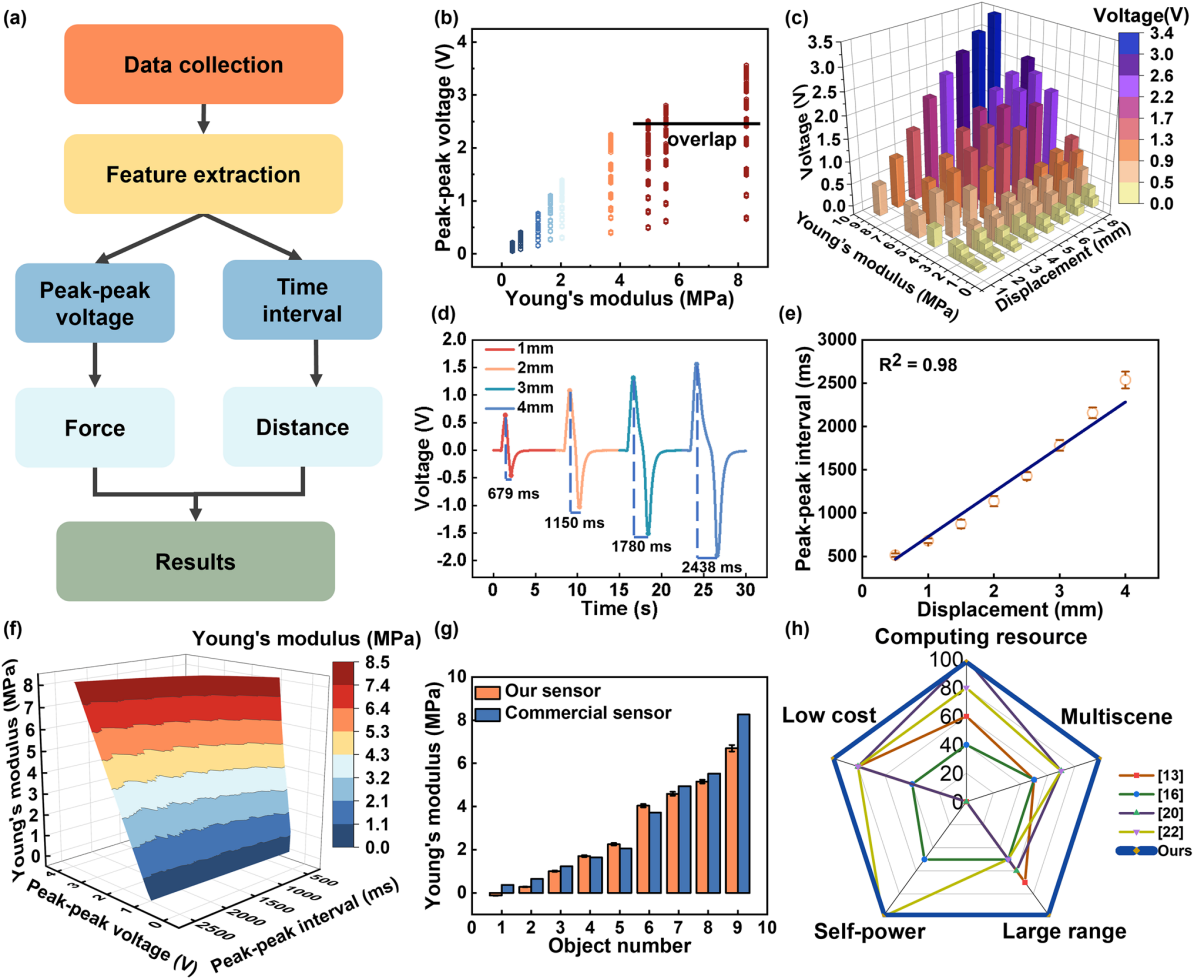
Signals decoupling for stiffness recognition. **a** Decoupling process of the measured signals. **b** Scatter diagram of the peak-peak voltage versus different stiffness. **c** Voltage of different objects versus different stiffness under known displacement. **d** Time interval between the positive peak value and the negative one under different displacement for Object 9. **e** Fitting relationship between time interval and displacement. **f** Young’s Modulus fitting curve generated from peak-peak voltage and time interval. **g** Young’s modulus measurement comparison between TENG and the commercial sensor. **h** Comparison between our sensor and other works

The process to decouple the stiffness of the pressed objects is to extract force and displacement from the Stiff-TENG output (Fig. 3a). The peak-peak voltage is an important characteristic of the Stiff-TENG output signal. The scatter diagrams in Fig. 3b display the peak-peak voltage for the nine objects. Solely relying on this feature makes it impossible to directly distinguish the stiffness, as specific values overlap between different objects. However, the stiffness can be identified under each specific displacement, as the harder object corresponds to the larger peak-peak voltage (Fig. 3c) and larger transferred charge (Fig. S12). Therefore, by extracting the displacement-related feature, the stiffness can be decoupled from the Stiff-TENG signals. However, the time interval between the positive and the negative peak (Fig. 3d) is relative to displacement. For details, when exerting force on an object, the voltage output reaches its positive peak once the object deformation reaches its maximum. After removing the pressure, the object rebounds. Since the object is limited by the equipment during the rebound, the motion damping during the rebound is relatively large. Thus, no over-bounce occurs. Once the object is completely restored to its original shape, the Stiff-TENG output voltage reaches the negative peak. Limited by the constant withdrawal speed and the overall displacement $\widehat{x}$, the peak-peak interval during one such process is solely related to $\widehat{x}$ (Fig. 3e, the details are shown in Fig. S13). Therefore, the overall displacement is obtained through the peak-peak interval. The peak-peak interval under different withdraw speed is shown in Fig. S14, indicating that the peak-peak interval is inversely proportional to the pressing speed. Then, by fitting the stiffness with the peak-peak interval and the peak-peak voltage (Fig. 3f), the recognition of object stiffness under arbitrary $\widehat{x}$ is realized.

To compare the performance of the Stiff-TENG with the results from the push–pull force gage, the results under 4 mm displacement are taken as one example. Both measurements (Fig. 3g) exhibit the same trend. Moreover, the self-powered Stiff-TENG offers several advantages over the other works. Firstly, the self-powered sensor has a lower cost (accounting is shown in Table S1), making it a more cost-effective option. Secondly, it requires fewer computing resources, simplifying the implementation process. Thirdly, it can measure the stiffness under large range displacement, making it possible to identify the deep-seated inclusions. Lastly, the Stiff-TENG can be utilized in a larger range of scenarios, providing more versatility (Fig. 3h). Furthermore, the durability of the Stiff-TENG is tested by pressing it for 3000 cycles (Fig. S15).

### Heterogeneous Stiffness Recognition and Encapsulated Objects Detection with the Stiff-TENG

**Fig. 4:**
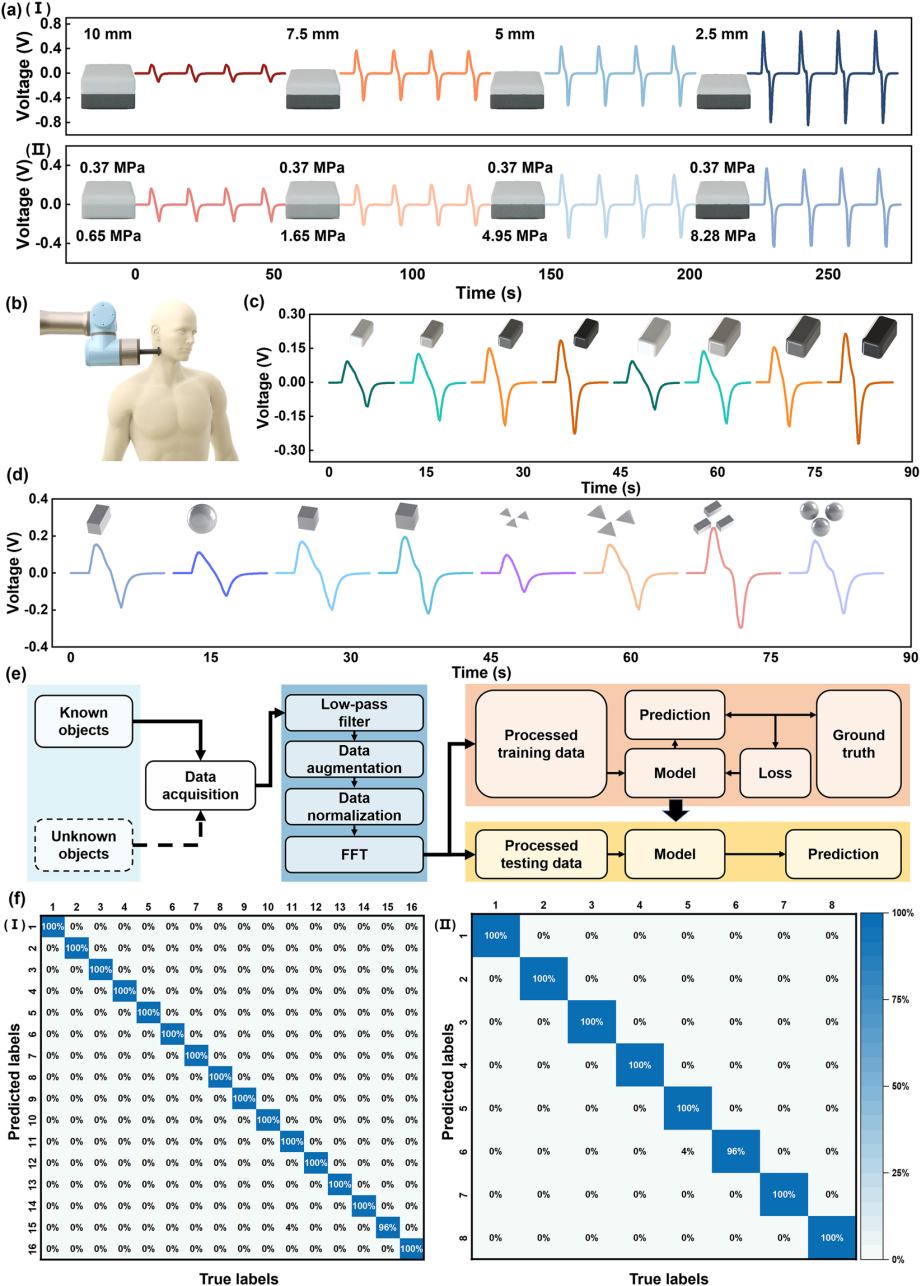
Heterogeneous stiffness characterization and inclusions detection. **a** Characteristic of the objects with different upper thickness and underneath stiffness. **b** The encapsulated objects detection illustration. **c, d** The output signals generated from pressing the objects with encapsulated different stiffness materials, and encapsulated 3D-printed materials of different sizes, shapes and amounts. **e** The block diagram of the data processing procedure of the heterogeneous stiffness application. **f** (I) The confusion matrix of the machine learning outcome for recognizing the 16 different 3D printed objects encapsulated. (II) The confusion matrix of the machine learning outcome for recognizing the soft object with encapsulated different stiffness

To characterize the nonuniformly distributed objects, double-layer soft objects with various upper layer thickness (2.5, 5.0, 7.5, and 10.0 mm) or bottom layer stiffness (0.65, 1.65, 4.95, and 8.28 MPa) are fabricated. The bottom layers’ thickness is the same (7.5 mm), and the upper layers’ stiffness is constant (0.37 MPa). The first scenario aims to explore the effect of varying the thickness of the upper layer while keeping the stiffness of the bottom layer constant. During the TENG pressing process, the device first comes into contact with the softer top layer, and the deformation of these three parts occurs simultaneously. It is known that Young’s modulus can be expressed as  $\varepsilon = \frac{F}{{\Delta l/l}}$,where ∆*l* and *l* denote the deformation length and original length. The thinner top layer deforms less than a thicker one under the same touching force. Meanwhile, the bottom layer of different samples deforms to a similar extent, so as the Stiff-TENG's elastic sponge layer. Therefore, the overall deformation which represents the displacement of the equipment tip is less for the sample with the thinner top layer. Thus, the Stiff-TENG deforms larger when press against the object with the thinner upper layer under the same overall displacement, resulting in a larger exhibited force as illustrated in Fig. 4aI. The second scenario considers objects with different stiffness for the bottom layer but the same upper layer thickness. The upper layer and the elastic sponge undergo the same forces and deformation under the same touching force. However, the bottom layer with various stiffness undergoes the same forces yet different levels of deformation, with harder objects deforming less according to Eq. (2). Therefore, under the same displacement, the object with a harder bottom layer undergoes larger force, resulting in greater elastic sponge deformation. This leads to a higher voltage level exhibited in the generated signal (Fig. 4aII).

The Stiff-TENG's ability to recognize objects with heterogeneous stiffness allows for its application in identifying materials encapsulated within objects. The Stiff-TENG is placed on the end of the robot arm via a solid rod. The Stiff-TENG can press different objects along with the movement of the robotic arm (Fig. 4b). When there are soft materials of different stiffness encapsulated in the soft object (Fig. S16a), the harder the inner part, the stronger resisting ability of the total structure resulting in a larger corresponding TENG voltage signal (Fig. 4c). When the Stiff-TENG presses the object encapsulating foreign object, the elastic sponge undergoes a continuous process of compression and recovery during the pressing operation. Consequently, the variations in force exerted by the pressed object against the sensor's compression become manifest in the waveform alterations of the output signal. Therefore, for encapsulated foreign objects, they affect the object's overall ability to resist deformation during the pressing to the same degree (Fig. S16b), regardless of their sizes, shapes, or numbers (Movie S2). The process of modal fabrication is illustrated in Fig. S17. Therefore, the encapsulated object distribution can be identified by analyzing the sensor response (shown in Fig. 4d, the details are in Fig. S18).

To identify the complex encapsulated objects inside, the collected data should be processed as shown in Fig. 4e. The data are segmented and signal components within 0–40 Hz is retained by low pass filter. To enhance the ability of the learning model to cope with real data, the data are augmented. The main step is to randomly move a segmented sample on the time axis to simulate the situation that the signal appears at any position in the acquisition queue during the actual data acquisition process (details are illustrated in Note S3). The data are then aligned according to the positive peak value. Afterward, the processed data are transformed into the frequency domain. The transformation to the frequency domain can satisfy the time translation invariance in the data processing process, and enhance the contained features. After that, the frequency domain data is divided into training set, verification set and test set according to the ratio of 4:3:3. The verification set is used to evaluate the performance of the algorithm during the training process. The test set is used to verify the performance of the algorithm on unknown data. Finally, the data in the frequency domain are used as input and processed using machine learning methods. Classic machine learning algorithm Support Vector Machine (SVM) illustrated in Note S4 is adopted and finally achieved 99.7% accuracy on the test set for the objects with hard inclusions (Fig. 4fI), and 99.5% accuracy on the test set for the objects with soft inclusions (Fig. 4fII).

## Conclusions

In this work, a self-powered stiffness sensor based on TENG with broad range of measurement is proposed. The Stiff-TENG consists of an ITO film, an FEP film coated with conductive ink printed electrode, and one holed elastic sponge. Two acrylic boards are attached to the FEP and the ITO film, respectively. Furthermore, a shielding film is attached to the bottom and connected to the ground. The Stiff-TENG can decouple the object’s stiffness regardless of the movement displacement in the range of 4 mm without the assistance of other sensors.

Due to Stiff-TENG’s ability to respond different pressing process, the characteristics for heterogeneous stiffness objects which are nonuniformly distributed is studied. Furthermore, the Stiff-TENG can be utilized for identifying objects that conceal different materials which are of various shapes, sizes, amounts and stiffness. The reason is that the foreign materials encapsulated within the object, regardless of their shapes, sizes, and amounts, alter the object's overall ability to resist deformation. With the processing pipeline made of data augmentation, normalization, FFT, and machine learning, the recognition accuracy achieves 99.7%. The generated signals can be displayed on the computer interface in real time. As it can recognize the objects whose encapsulated structure exhibits similar characteristics to those found in soft tissue lesions or cancerous growths in the human body, the Stiff-TENG has significant potential for the advancement of self-powered palpation sensing.

## Supplementary Information

Below is the link to the electronic supplementary material.

Supplementary file 1 (MP4 9075 KB)

Supplementary file 2 (MP4 9985 KB)

Supplementary file 3 (PDF 1556 KB)
