# Correction: A Broad Range Triboelectric Stiffness Sensor for Variable Inclusions Recognition

**DOI:** 10.1007/s40820-024-01614-y

**Published:** 2025-01-14

**Authors:** Ziyi Zhao, Zhentan Quan, Huaze Tang, Qinghao Xu, Hongfa Zhao, Zihan Wang, Ziwu Song, Shoujie Li, Ishara Dharmasena, Changsheng Wu, Wenbo Ding

**Affiliations:** 1 Tsinghua-Berkeley Shenzhen Institute, Institute of Data and Information, Shenzhen International Graduate School, Tsinghua University, Shenzhen, 518055 People’s Republic of China; 2 Institute of Ocean Engineering, Shenzhen International Graduate School, Tsinghua University, Shenzhen, 518055 People’s Republic of China; 3 Wolfson School of Mechanical Electrical and Manufacturing Engineering, Loughborough University, Loughborough, LE11 3TU UK; 4 Department of Materials Science and Engineering, National University of Singapore, Singapore, 117575 Singapore; 5 RISC-V International Open Source Laboratory, 518055 Shenzhen, People’s Republic of China

**Correction to: Nano-Micro Lett. (2023) 15:233** 10.1007/s40820-023-01201-7

Following publication of the original article [1], the authors reported that the first two lines of the introduction were accidentally placed in the right-hand column of the page in the PDF, which affects the readability.

The original article [1] has been corrected.

## References

[1] Z. Zhao, Z. Quan, H. Tang et al., A Broad Range Triboelectric Stiffness Sensor for Variable Inclusions Recognition. Nano-Micro Lett. **15**, 233 (2023). 10.1007/s40820-023-01201-7 10.1007/s40820-023-01201-7 PMC10589179 37861802

