# Supplementary material for: A Broad Range Triboelectric Stiffness Sensor for Variable Inclusions Recognition
Source: Nanomicro Lett. 2023 Oct 20;15:233. doi: 10.1007/s40820-023-01201-7 (PMC10589179; doi:10.1007/s40820-023-01201-7)
Supplement: Supplementary file 3 — (PDF 1556 KB) [file 40820_2023_1201_MOESM3_ESM.pdf]

Supporting Information for

A Broad Range Triboelectric Stiffness Sensor for Variable Inclusions Recognition

Ziyi Zhao[1, #], Zhentan Quan[2, #], Huaze Tang[1, #], Qinghao Xu[1], Hongfa Zhao[1], Zihan Wang[1] ,Ziwu Song[1], Shoujie Li[1], Ishara Dharmasena[3], Changsheng Wu[4], and Wenbo Ding[1,5*]

[1] Tsinghua-Berkeley Shenzhen Institute, Institute of Data and Information, Shenzhen International Graduate School, Tsinghua University, Shenzhen, 518055, People's Republic of China

[2] Institute of Ocean Engineering, Shenzhen International Graduate School, Tsinghua University, Shenzhen, 518055, People's Republic of China

[3] Wolfson School of Mechanical Electrical and Manufacturing Engineering, Loughborough University, Loughborough, LE11 3TU, UK

[4] Department of Materials Science and Engineering, National University of Singapore, Singapore, 117575, Singapore

[5] RISC-V International Open Source Laboratory, 518055, Shenzhen, People's Republic of China

#Ziyi Zhao, Zhentan Quan, and Huaze Tang contributed equally to this work

*Corresponding author. E-mail: ding.wenbo@sz.tsinghua.edu.cn (Wenbo Ding)

Supplementary Figures and Tables

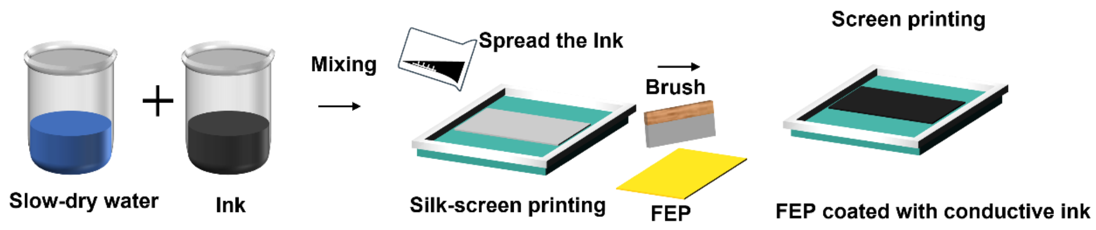

Fig. S1 The manufacturing process of the conductive ink attached to the FEP film. **a** Mix and stir the conductive ink (CH-8(MOD2) and the slow-dry water at a ratio of 1:4. **b** Place the back side of the FEP film facing up, place the mesh screen printing plate above the FEP film, pour a suitable amount of mixture onto the mesh screen printing plate, and quickly push the mixture from one end of the plate to the other end using a brush. **c** Place the printed FEP film at room temperature for 24 hours

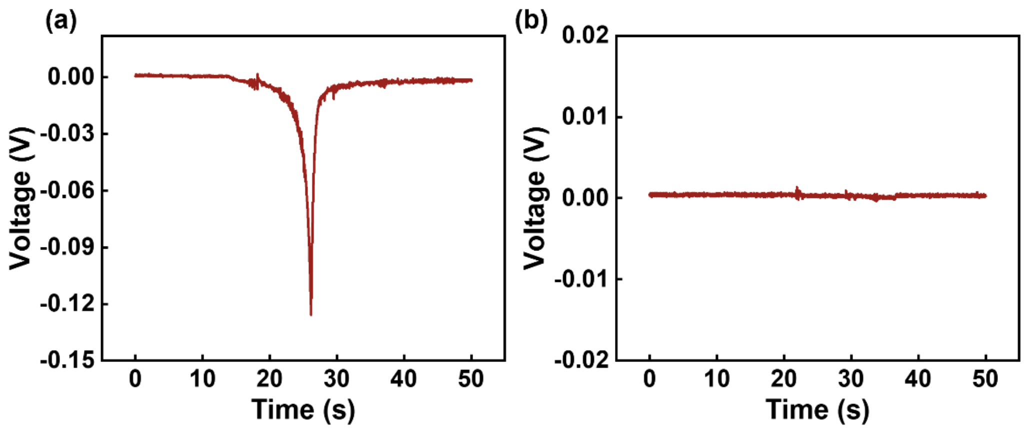

Fig. S2 The voltages generated when Stiff-TENGs with different structures approach the object. **a** The voltage signal generated when the Stiff-TENG without shielding layer approaches the object. **b** The voltage signal generated when the Stiff-TENG approaches the object

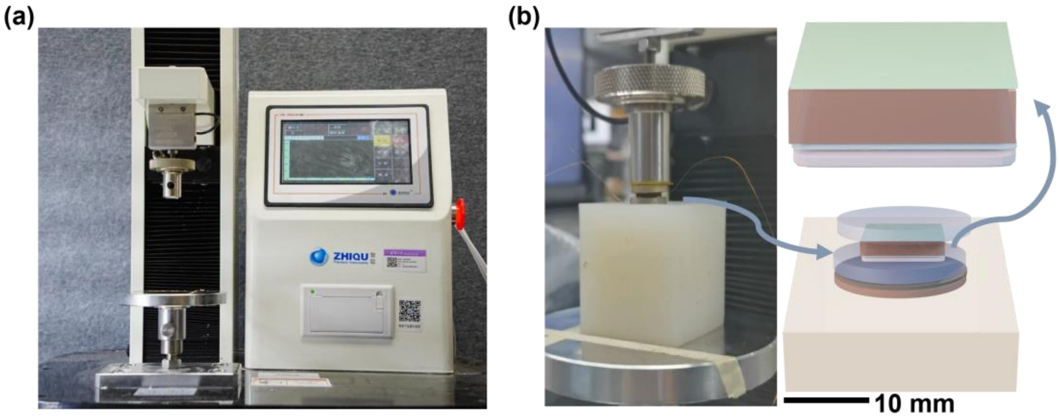

Fig. S3 a Photograph of the standardize experiment platform. **b** The experiment platform details for the standard calibration and data collection

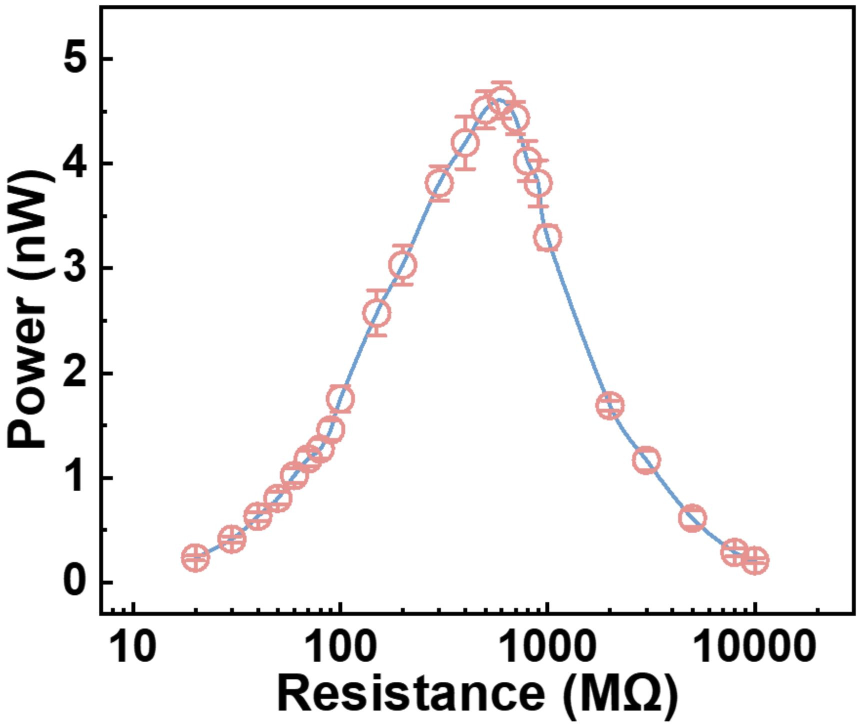

Fig. S4 The output power under different external load resistance

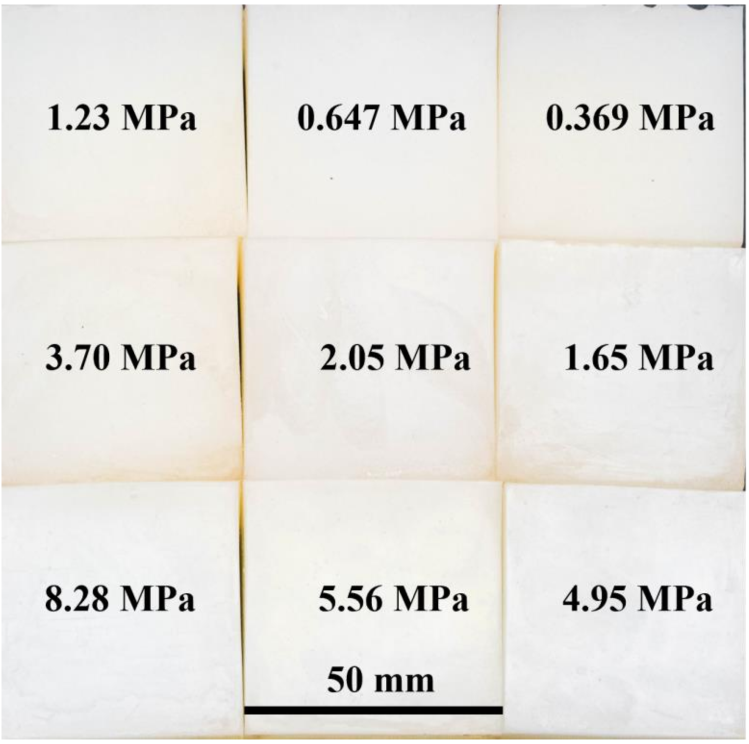

Fig. S5 The standardize objects with different stiffness ranging from 0.37 MPa to 8.28 MPa

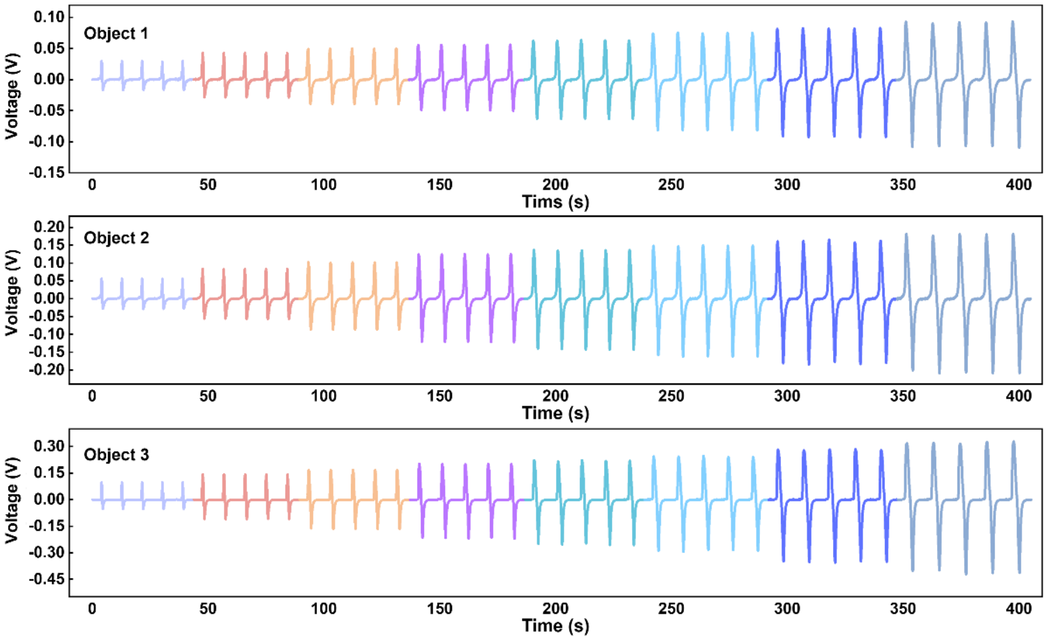

Fig. S6 The output voltage of the Stiff-TENG under the displacement ranging from 0 mm to 4 mm when pressing the Object 1-3

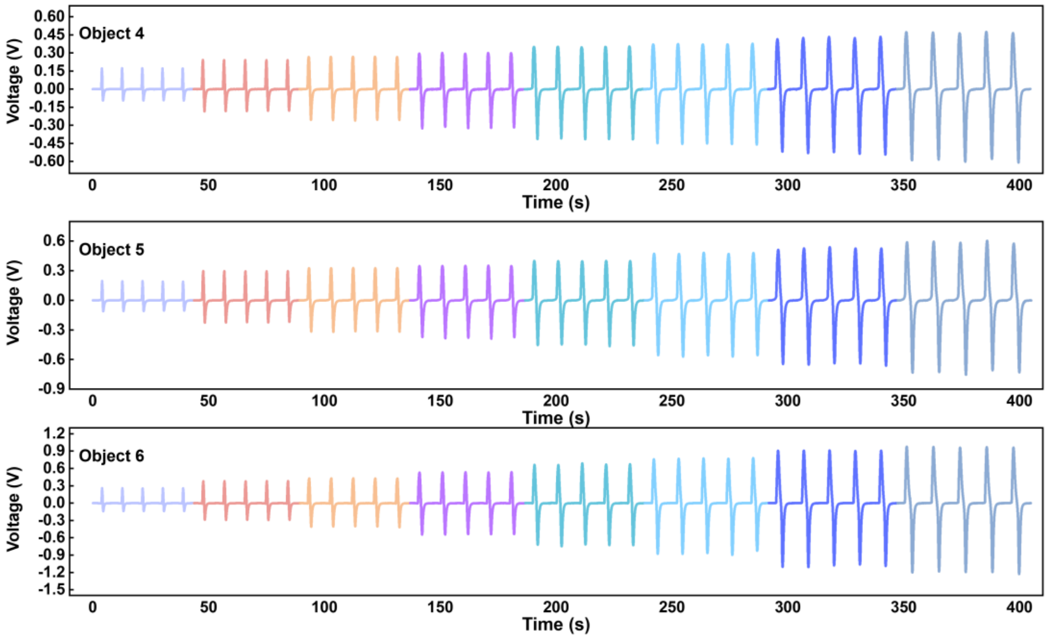

Fig. S7 The output voltage of the Stiff-TENG under the displacement ranging from 0 mm to 4 mm when pressing the Object 4-6

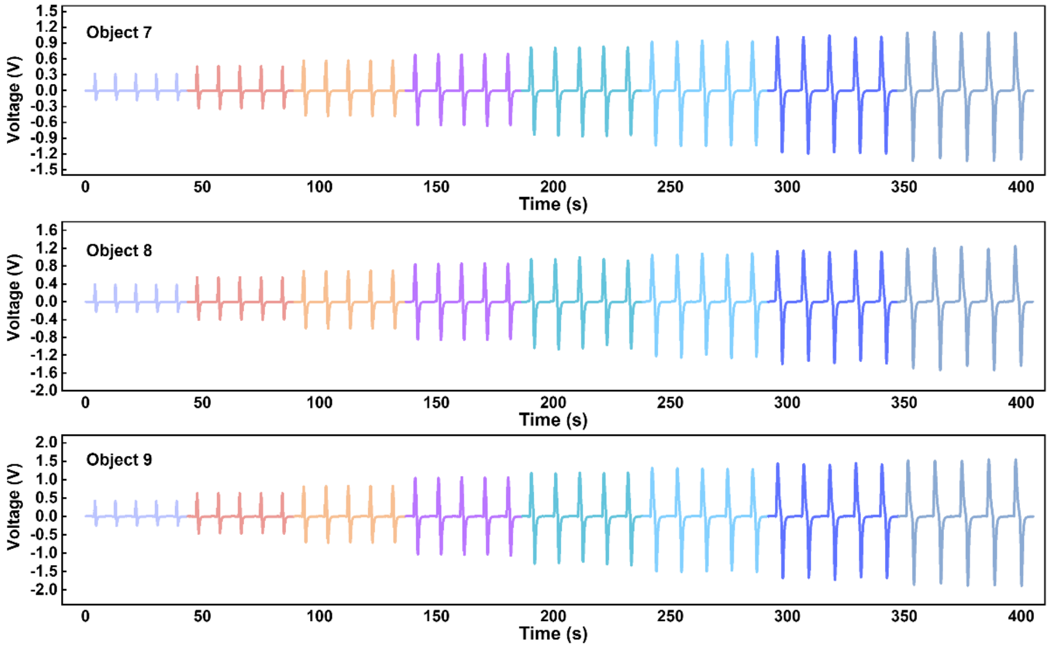

Fig. S8 The output voltage of the Stiff-TENG under the displacement ranging from 0 mm to 4 mm when pressing the Object 7-9

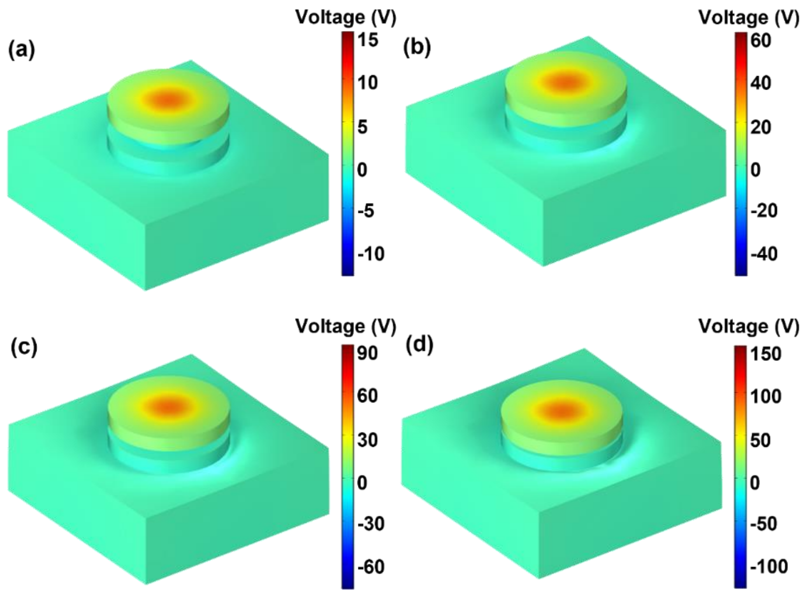

Fig. S9 COMSOL simulation of electric potential distribution under different pressing level. From **a** to **d**, the Stiff-TENG presses the same object with increasing upper acrylic displacement extent

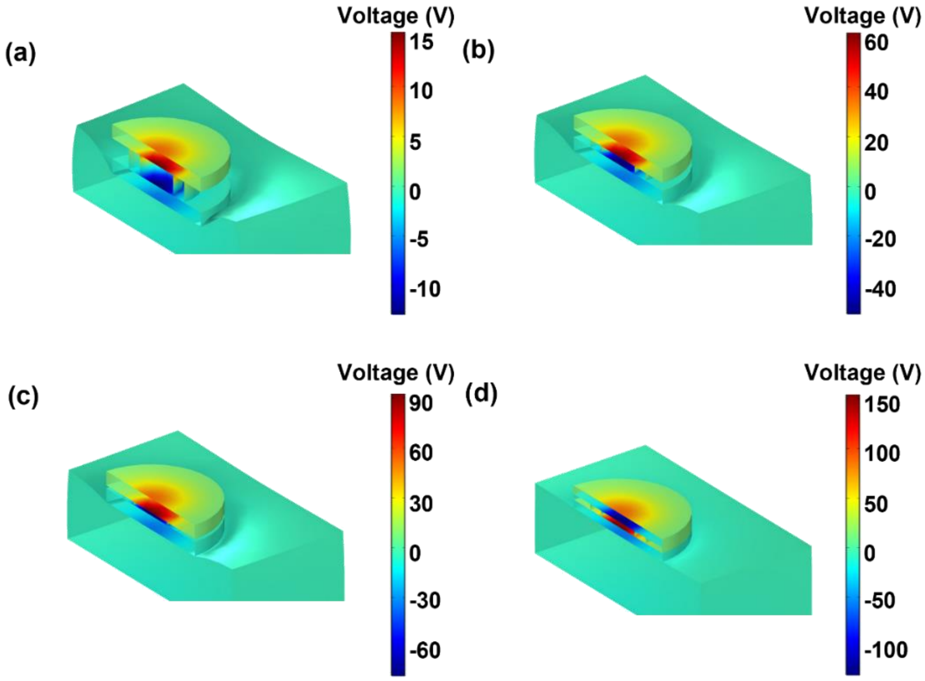

Fig. S10 COMSOL simulation of electric potential distribution under the same total displacement. From **a** to **d**, the S-TENG presses the objects with increasing stiffness

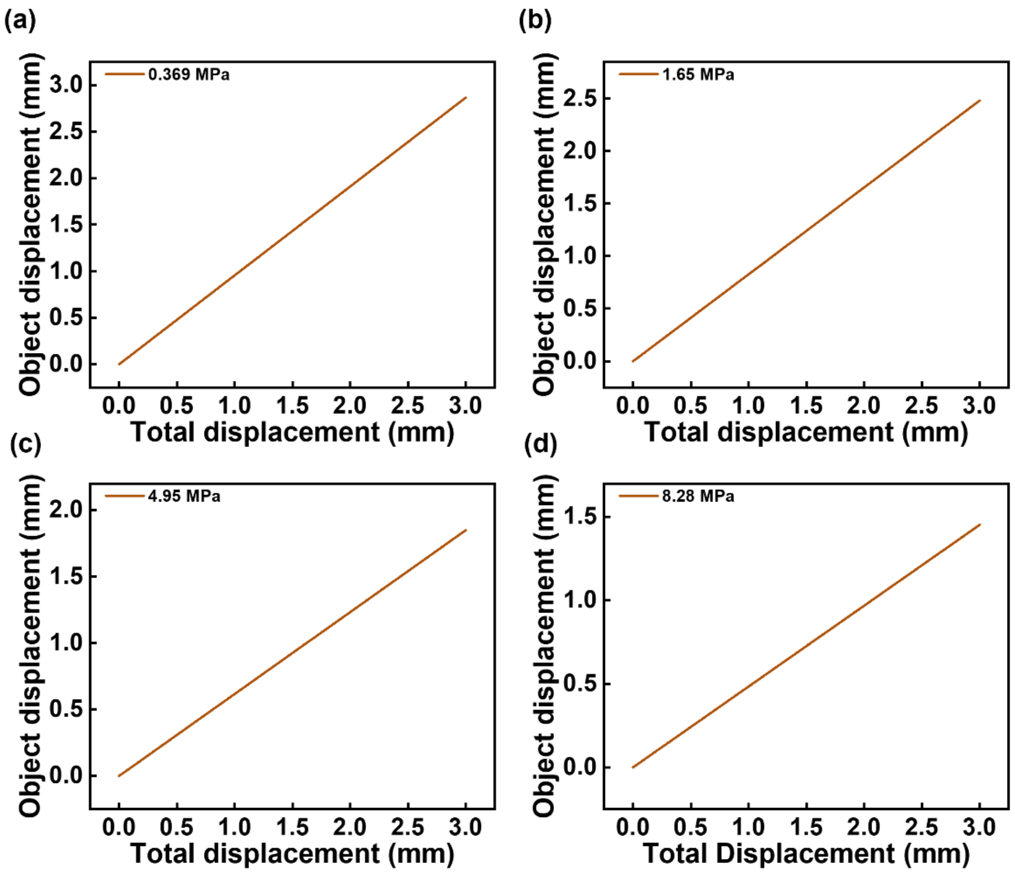

Fig. S11 COMSOL simulation of the different objects' deformation value under the same overall deformation level

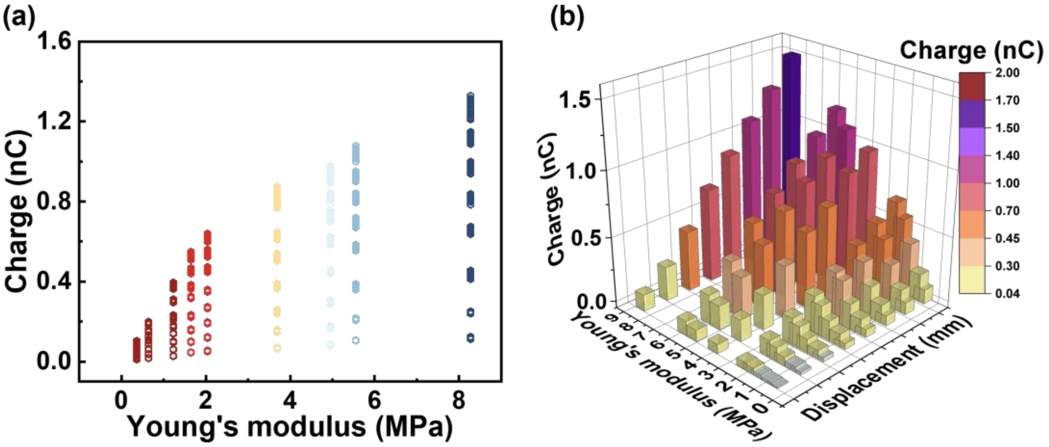

Fig. S12 a The scatter diagram of the transfered charges versus different stiffness objects. **b** The transfered charges of different objects versus different stiffness under known displacement

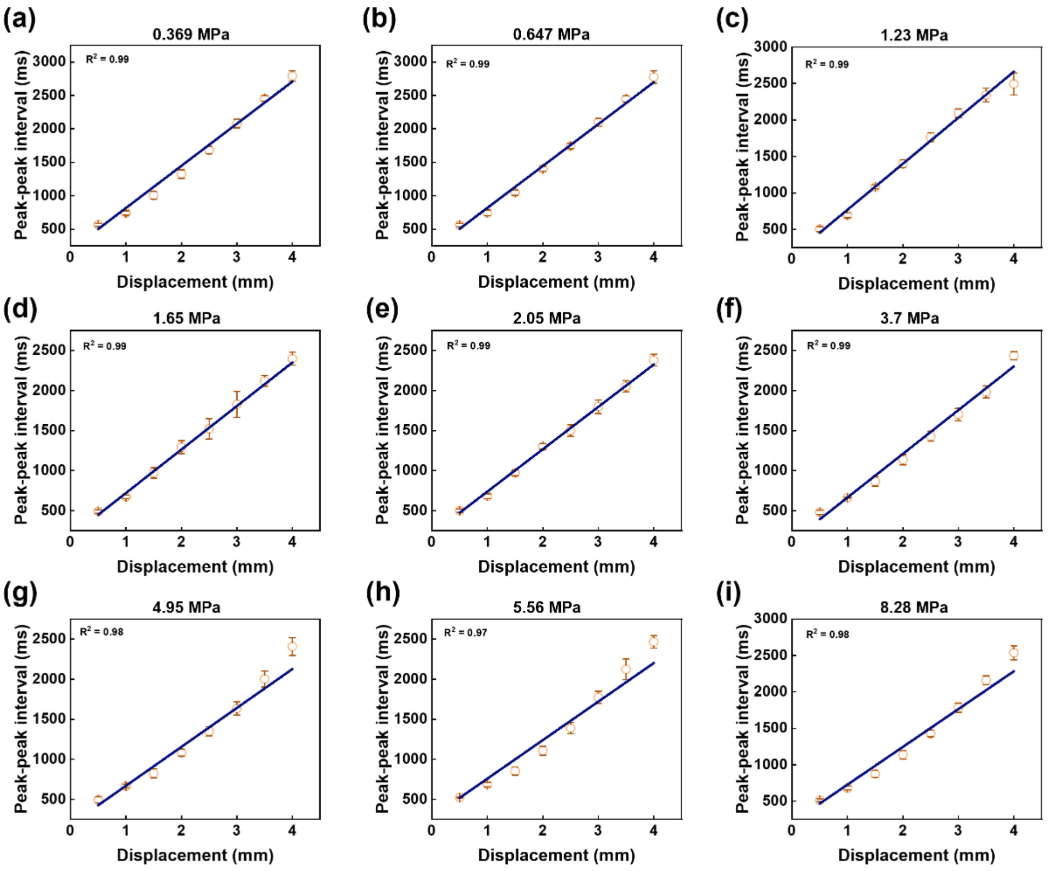

Fig. S13 The time interval between the positive and negative peaks of one signal period when pressing nine objects with different stiffness levels (ranging from 0.369 MPa to 8.28 MPa from **a** to **i** at various displacements (ranging from 0 mm to 4 mm)

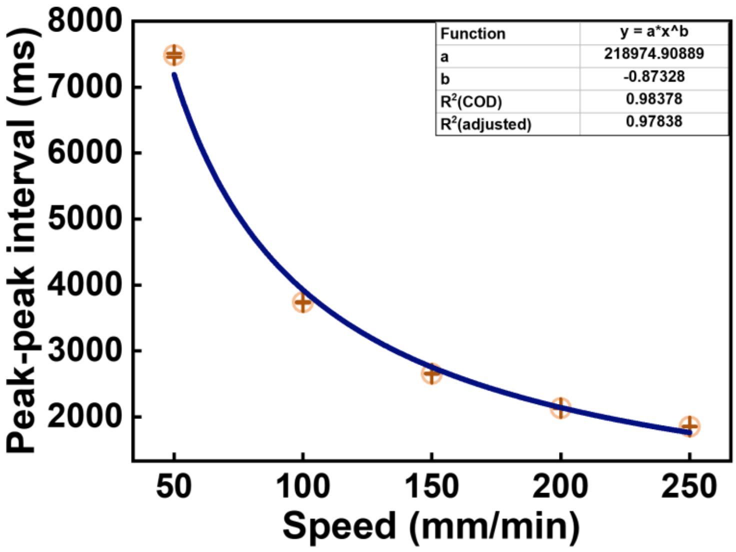

Fig. S14 The time intervals between the positive peak and the negative one under different speed pressing

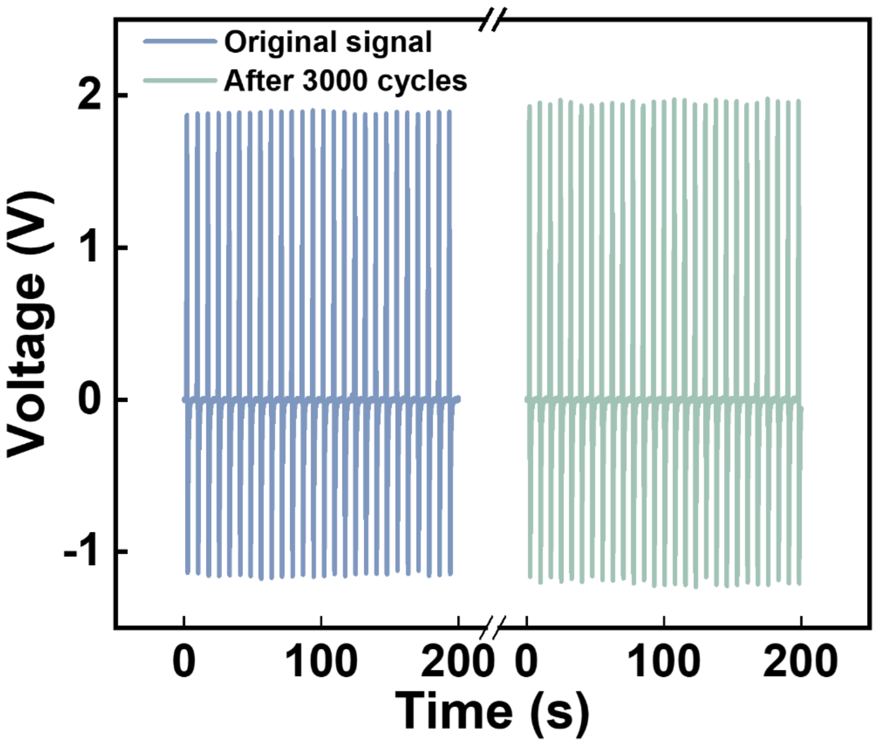

Fig. S15 Comparison of the original voltage and that after working 3000 cycles from the Stiff-TENG

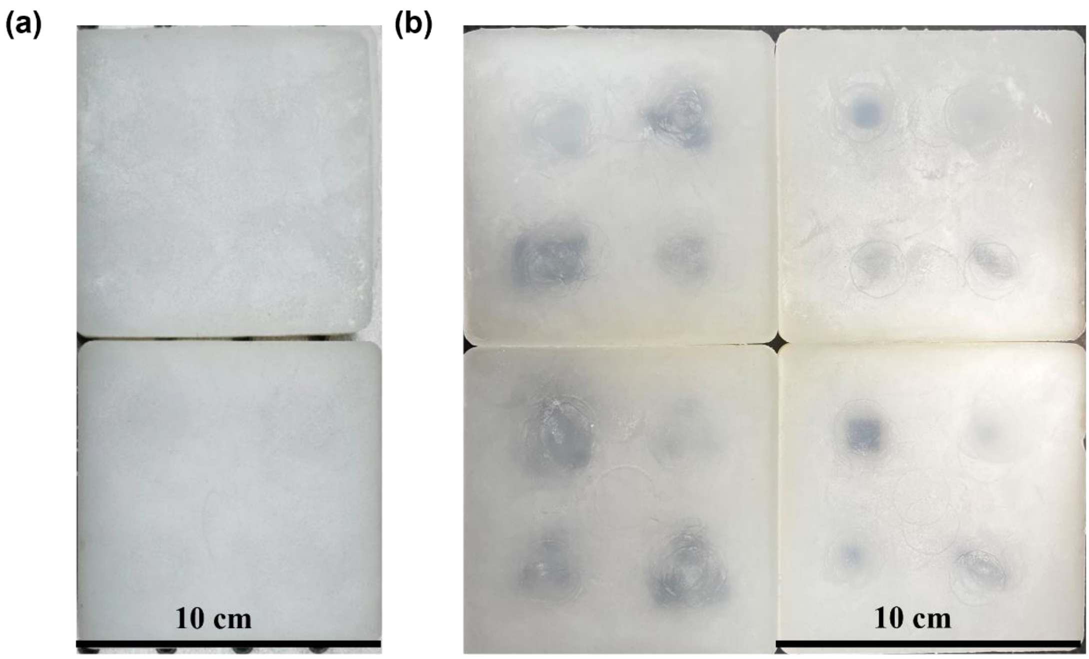

Fig. S16 a Objects of different sizes and stiffness encapsulated within silicon rubber. **b** 3D printed objects in different shapes, sizes, and numbers concealed within silicon rubber

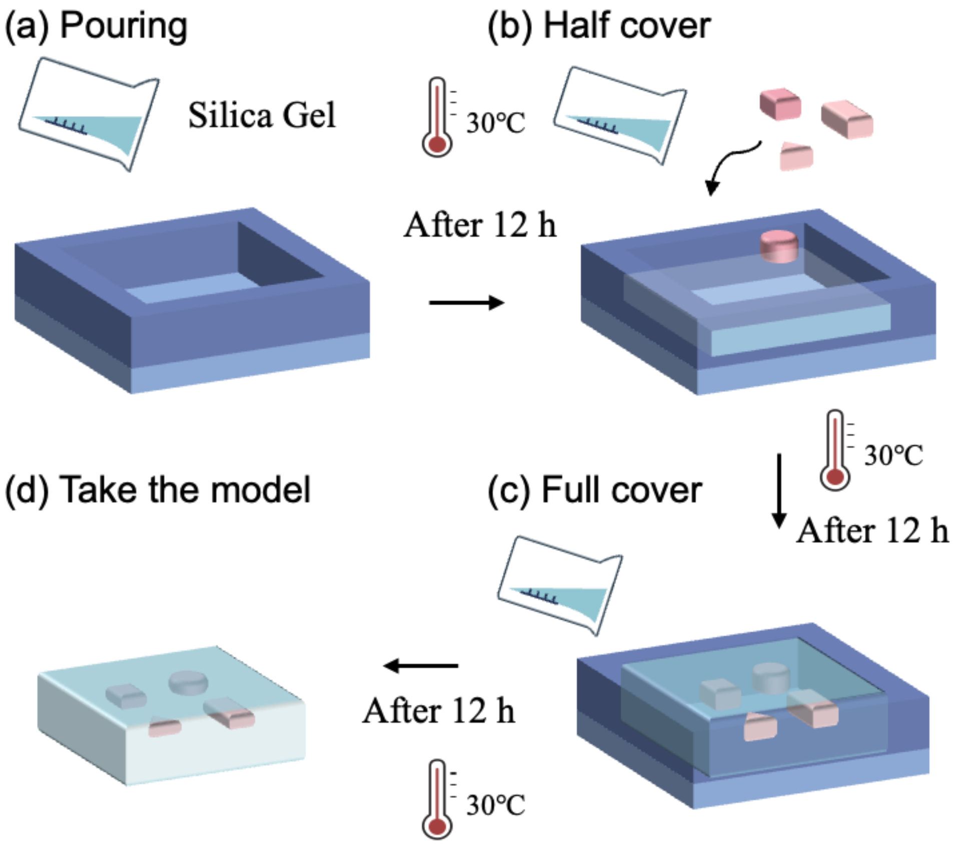

Fig. S17 Fabrication process of the modle**. a** Pour the mixed silica gel into the 3D printed container to a height of 7.5 mm. **b** After 12 hours heating, place different 3D printed objects or poured soft blocks on the solidified silica gel, and pour an appropriate amount of silica gel to cover half of the blocks. **c** After another 12 hours heating, pour the mix silica gel until the whole height reaches 15 mm. **d** After the last heating, the shaped model is released from the mold

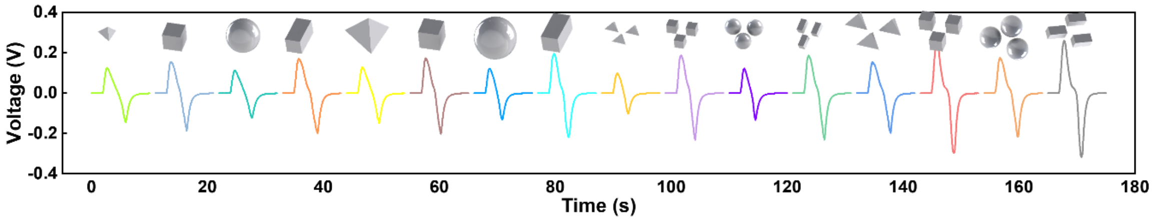

Fig. S18 Voltage generated by the Stiff-TENG from pressing soft materials with 16 kinds of encapsulated objects of different sizes, shapes, and amounts

Note S1: Shielding film

When an object is connected to a grounded metal surface, it allows for the transfer of electric charge between the object and the ground. By grounding an object, any excess charge on the surface of the object can be dissipated into the ground, effectively neutralizing the charge and reducing the potential for electrical interference.

The process of grounding can be mathematically described using the principles of electrostatics. According to Coulomb's law, the force between two electrically charged objects is proportional to the product of their charges and inversely proportional to the square of the distance between them. Therefore, when an object is grounded, the electric charge on its surface is distributed across the ground, reducing the concentration of charge on the object's surface. This effect can be quantified using the capacitance equation: $C = \varepsilon A/d$, where C is the capacitance of the object, ε is the permittivity of the surrounding medium, A is the area of the object's surface, and d is the distance between the object's surface and the ground.

Note S2 Manufacturing Method and Surface De-adhesion Treatment of Different Stiffness Standardize Silicone Blocks

To produce standardized blocks with different stiffness, different type of silicone gel was used. For each stiffness of silicone gel, an equal mass of silicone gel A and B were weighed using a plastic container, and the mixture was thoroughly stirred with a spatula for 1-2 minutes to ensure uniform mixing. The container with the mixture was then placed in a vacuum machine and evacuated for 3 minutes before pouring it into a square mold with a side length of 50mm. Finally, the mold was placed in an oven and heated at a low temperature of 30° C for 12 hours to obtain a standard block.

The soft silicone blocks produced have a high surface adhesion, which can contaminate the experimental equipment and be inconvenient for use in experiments. To overcome this problem, the manufactured blocks were first soaked in alcohol for one hour, then removed, dried, and placed in a UV irradiation device for 2 hours. Finally, Johnson's baby powder was applied to the surface of the blocks to obtain a standard silicone cube with a skin-like touch.

Note S3 Data Augmentation

For dataset $D = (x_1, y_1), (x_2, y_2), \ldots, (x_n, y_n)$, where x_i is the input data and y_i is the corresponding label, translating the data can enhance the robustness of the dataset. Specifically, for each data point (x_i, y_i) ,generate k new data points $(x_{i,1}, y_i), (x_{i,2}, y_i), \ldots, (x_{i,k}, y_i)$ such that these new data points correspond to the original data point (x_i, y_i) but are located slightly differently in the sample space.

The translation process involves cyclically shifting each original data point(x_i, y_i)on the time axis by$d_1, d_2, \ldots, d_k$units, where $d_j$is a vector representing the time difference for the$j$th shift. Then for each translation vector d_j a new data point $(x_{i,j}, y_i)$ is generated, where $x_{i,j} = x_i + d_j$.

To generate these translation vectors d_j, k vectors are randomly selected from a predefined set of translation vectors $\mathcal{D} = d_1, d_2, \ldots, d_m$. Specifically, for each original data point (x_i, y_i), randomly select k translation vectors from $\mathcal{D}$ and use them to generate k new data points $(x_{i,1}, y_i), (x_{i,2}, y_i), \ldots, (x_{i,k}, y_i)$.

The principle behind data translation is to move data points from their original positions to new positions. Suppose the coordinates of the original data point are (x_i^0, y_i^0) and the translation vector is d, then the new coordinates after translation are $(x_i, y_i) = (x_i^0 + d, y_i^0)$. This can be viewed as an affine transformation of the input space of the original data point because the translation vector d can be seen as an affine transformation matrix that shifts points in the input space along a specific direction by a specific distance. This transformation can enhance the dataset and improve the generalization performance of the model.

Note S4 SVM principle

For a labeled dataset, where each data point is represented by x_i and its corresponding label is $y_i \in -1{,}1$, the goal of SVM is to find a hyperplane $w^T x + b = 0$, where w is the normal vector and b is the bias term, that can separate the data points into two classes and maximize the margin between the hyperplane and the nearest data points. The distance from a data point to the hyperplane can be calculated using the following formula $\frac{1}{\|w\|}|w^T x_i + b|$,where $\| w \|$ is the L2 norm of w.

We aim to minimize the number of misclassifications and maximize the margin between the hyperplane and the nearest data points. This can be formulated as a convex optimization problem:

$$\min_{w,b}\frac{1}{2}\parallel w\parallel^2 \text{ s.t. } y_i(w^Tx_i+b)\geq 1,\forall i$$

where$\parallel w\parallel^2$ is the objective function want to minimize, and $y_i(w^Tx_i+b)\geq 1$ is the constrain that ensures each data point is correctly classified.

Since the optimization problem is convex, existing optimization algorithms such as Lagrange duality can be used to solve it. Additionally, to handle nonlinear classification problems, we can use kernel functions to map the original data into a high-dimensional space.

Finally, the strategy for SVM to select the hyperplane is to find the hyperplane that maximizes the classification margin, which can be represented as:

$$w^Tx+b=0$$

where w is the normal vector and b is the bias term, both of which can be computed from the training dataset.

Table S1 Bill of materials for the S-TENG fabrication

Description	Cost
1 cm^2 FEP film	< US$0.01
1 cm^2 ITO film	< US$0.01
4.54 cm^2 Acrylic board	< US$0.01
2.27 cm^2 Kapton film	< US$0.01
1 cm^2 Elastic sponge (2mm)	< US$0.01
2.27 cm^2 Elastic sponge (1mm)	< US$0.01
2.27 cm^2 Shielding film	< US$0.01
30 cm Copper wire	< US$0.01
**Total:**	**< US$0.01**
